# Bufei Yishen Granules Combined with Acupoint Sticking Therapy Suppress Inflammation in Chronic Obstructive Pulmonary Disease Rats: Via JNK/p38 Signaling Pathway

**DOI:** 10.1155/2017/1768243

**Published:** 2017-10-30

**Authors:** Yange Tian, Ya Li, Jiansheng Li, Suxiang Feng, Suyun Li, Jing Mao, Yang Xie, Xuefang Liu, Haoran Dong, Wanchun Zheng, Minghang Wang

**Affiliations:** ^1^Henan Key Laboratory of Chinese Medicine for Respiratory Disease, Henan University of Chinese Medicine, Zhengzhou, Henan 450046, China; ^2^Collaborative Innovation Center for Respiratory Disease Diagnosis and Treatment & Chinese Medicine Development of Henan Province, Henan University of Chinese Medicine, Zhengzhou, Henan 450046, China; ^3^Central Laboratory and Respiratory Pharmacological Laboratory of Chinese Medicine, The First Affiliated Hospital, Henan University of Chinese Medicine, Zhengzhou, Henan 450008, China; ^4^Institute of Respiratory Diseases, The First Affiliated Hospital, Henan University of Chinese Medicine, Zhengzhou, Henan 450008, China; ^5^Institute of Pharmacy, Henan University of Chinese Medicine, Zhengzhou, Henan 450046, China

## Abstract

The present study was initiated to explore the mechanism of the effects of Bufei Yishen granules combined with acupoint sticking therapy (Shu-Fei Tie) on inflammation regulated by c-Jun N-terminal kinase (JNK) and p38 MAPK signaling in COPD rats. Seventy-two rats were divided into healthy control (Control), Model, Bufei Yishen (BY), acupoint sticking (AS), Bufei Yishen + acupoint sticking (BY + AS), and aminophylline (APL) groups (*n* = 12 each). COPD rats were exposed to cigarette smoke and bacteria and were given the various treatments from weeks 9 through 20; all animals were sacrificed at the end of week 20. MCP-1, IL-2, IL-6, and IL-10 concentrations in BALF and lung tissue as well as JNK and p38 mRNA and protein levels in lung were measured. The results showed that all the four treatment protocols (BY, AS, BY + AS, and APL) markedly reduced the concentrations of IL-2, IL-6, and MCP-1 and levels of JNK and p38 MAPK mRNA, and the effects of Bufei Yishen granules combined with acupoint sticking therapy were better than acupoint sticking therapy only and aminophylline. In conclusion, the favorable effect of Bufei Yishen granules combined with Shu-Fei Tie may be due to decreased inflammation through regulation of the JNK/p38 signaling pathways.

## 1. Introduction

Chronic obstructive pulmonary disease (COPD) is a common, preventable, and treatable disease that is characterized by persistent respiratory symptoms and airflow limitation that is due to airway and/or alveolar abnormalities usually caused by significant exposure to noxious particles or gases [1]. COPD has been the third leading cause of death in China in 2010, behind only stroke and ischemic heart disease [2].

Inhalation of cigarette smoke or other noxious particles, such as smoke from biomass fuels, causes lung inflammation. The chronic inflammation response may induce parenchymal tissue destruction (resulting in emphysema) and disruption of normal repair and defense mechanisms (resulting in small airway fibrosis) [1]. The c-JUN N-terminal kinases (JNK) and p38 mitogen-activated protein kinase (MAPK) signaling, the main components of MAPK pathway, are closely correlated with the inflammatory response. JNK and p38 MAPK pathway can be activated by environmental stimuli, such as tobacco smoke, and by endogenous signals, such as cytokines, growth factors, and inflammation-derived oxidants. Recent studies have suggested that activation of the MAPK pathway contributes to several COPD-associated phenotypes, including mucus overproduction and secretion, inflammation, and cytokine expression [3]. Inflammatory mediators and chemotactic factors, including tumor necrosis factor-*α* (TNF-*α*), interleukin- (IL-) 6, and IL-10, which is mediated in part by the p38 MAPK pathway, contribute to the formation of pulmonary emphysema [4, 5]. TNF-*α* increased the expression of monocyte chemoattractant protein-1 (MCP-1) at least partly via enhancing phosphorylation of p38 and JNK [6]. Several studies have shown that therapies, such as treatment with bone marrow-derived mesenchymal stem cell (MSCs) and Panax ginseng (Ren Shen), may relieve airway inflammation and emphysema via the MAPK pathway [7, 8].

In recent years, Traditional Chinese Medicine (TCM) therapies, including internal and external treatments, have played an increasingly important role in stable COPD because of their favorable curative effect and few side effects [9, 10]. The pattern of lung-kidney qi deficiency, one type of TCM syndromes, is one of the most common syndromes in the stable phase of COPD [11]. Many different factors, such as cigarette smoking and noxious particles, may lead to lung qi weakness, and the patients will present dyspnea, shortness of breath, weakness, and spontaneous perspiration (worse with exertion), then kidney qi is damaged and becomes weak over time, and the patients will also present tinnitus, vertigo, frequent micturition, frequent urination at night, soreness, and weakness of the waist and knees. Bufei Yishen granules (ZL.201110117578.1), a special prescription for internal treatment of lung-kidney qi deficiency syndrome, were clinically proved effective in relieving clinical symptoms and reducing the frequency of acute exacerbations in stable COPD patients [12]. Additionally, Bufei Yishen granules were also confirmed effective in ameliorating systemic and airway inflammation and remodeling in a cigarette smoke/bacterial exposure-induced COPD rat model and preventing COPD and its comorbidities, such as ventricular hypertrophy [13–15]. Shu-Fei Tie (ZL.200810049332.3) is a popular clinically used ointment for acupoint sticking in external therapy which can excite vital qi in the human body and has been proven therapeutic in COPD treatment with its high safety, convenience, and fewer side effects [16, 17]. Our previous study has shown that Bufei Yishen granules combined with Shu-Fei Tie can alleviate clinical symptoms, reduce the frequency and duration of acute exacerbation, and improve lung function and quality of life in patients with stable COPD and also showed beneficial effect in a 4-month treatment period and 6 months of follow-up [18]. Our previous animal experimental study has also shown that this approach improves pulmonary function and lung pathological impairment in COPD rats [19]. We also found that this therapy can suppress oxidative stress in COPD rats [20].

However, whether or not it can suppress inflammation in COPD rats remains unclear. We want to know whether the effect of Bufei Yishen granules combined with Shu-Fei Tie is related to anti-inflammation. Thus, our current study was performed to examine the mechanism of Bufei Yishen granules combined with Shu-Fei Tie therapy on inflammation regulated by JNK and p38 MAPK signaling in COPD rats.

## 2. Materials and Methods

### 2.1. Animal Model

Seventy-two Sprague-Dawley rats (equal number of males and females, 2 months old, 180–220 g) were obtained from the Laboratory Animal Center of Henan Province (Special Pathogen Free, SCXK [Henan] 2010-0002) and randomly assigned to the Control, Model, Bufei Yishen (BY), acupoint sticking (AS), Bufei Yishen + acupoint sticking (BY + AS), and aminophylline (APL) groups (12 in each group). The methods were performed according to the approved guidelines of the Experimental Animal Care and Ethics Committee of the First Affiliated Hospital, Henan University of Traditional Chinese Medicine, Zhengzhou, China.

After accommodating to the facility for 7 days, COPD rats were exposed to cigarette smoke and* Klebsiella pneumoniae* (KP) for model establishment according to previously described methodology [21]. Commercial cigarettes (Hongqiqu® Filter Cigarette, Henan, China) were provided by Henan Tobacco Industry Co., Ltd., and each of these cigarettes contained 1.0 mg nicotine, 11 mg CO, and 10 mg tar oil, according to the manufacturer's specifications.* Klebsiella pneumoniae* (strain: 46114) was purchased from the National Center For Medical Culture Collection (Beijing, China) and prepared at a concentration of 6 × 10^8^ colony forming units (CFU) per milliliter before being administered to the animals. Animals were exposed to smoke (smoke concentrations, 3,000  ±  500 ppm) for 30 min, twice a day for 12 weeks.* Klebsiella pneumoniae* solution (0.1 ml, 6 × 10^8^ colony forming units/ml) was dropped into the two nostrils in an alternate fashion, once every 5 days, for the first 8 weeks. The successful generation of a COPD rat model was evaluated according to symptoms, lung function, and pulmonary pathology [22].

### 2.2. Drugs

(1) Aminophylline tablets (Xinhua, Shandong, China, 0.1 g/tablet) were crushed prior to administration to the animals. (2) Bufei Yishen granules [Ren Shen (Ginseng Radix et Rhizoma) 9 g, Huang Qi (Astragali Radix) 15 g, Shan Zhu Yu (Corni Fructus) 12 g, Yin Yang Huo (Epimedii Herba) 9 g, Gou Qi Zi (Lycii Fructus) 12 g, and Wu Wei Zi (Schisandrae Chinensis Fructus) 9 g etc.] were prepared by the Department of Pharmacology in the First Affiliated Hospital of Henan University of Traditional Chinese Medicine, Zhengzhou, China. (3) Shu-Fei Tie mainly consisted of Bai Jie Zi (Semen Brassicae) 10 g, Yan Hu Suo (Rhizoma Corydalis) 5 g, Xi Xin (Asarum Heterotropoides) 5 g, and Yuan Hua (Daphne Genkwa) 10 g and also included other components, 3.0 g/tubes. The main chemical compounds of Bufei Yishen granules and Shu-Fei Tie had been described in our published article [20]. The main component of Shu-Fei Tie placebo was carbopol, diatomaceous earth, and glycerine, each unit equivalent to 3.0 g. The placebo was also similar to the true drug in its appearance, weight, color, and odor. Shu-Fei Tie and its placebo were produced and packed by the Department of Pharmacology in the First Affiliated Hospital of Henan University of TCM, which was the reform base of TCM preparation and dosage formulation.

### 2.3. Administration

From weeks 9 through 20, rats in the Control and Model groups were intragastrically given normal saline (2 ml/animal, b.i.d) and Shu-Fei Tie placebo (2 times/week); Bufei Yishen granules (4.44 g/kg·d, b.i.d) and Shu-Fei Tie placebo were given to the BY group; normal saline and Shu-Fei Tie were given to the AS group; Bufei Yishen granules (4.44 g/kg·d, b.i.d) and Shu-Fei Tie were given to the BY + AS group, and aminophylline (2.3 mg/kg·d, b.i.d) and Shu-Fei Tie placebo were given to the APL group. Dosage adjustments were made weekly according to body mass. The equivalent dosages were calculated by using the following formula: *D*_rat_ = *D*_human_ × (*I*_rat_/*I*_human_) × (*W*_rat_/*W*_human_)^2/3^. *D*: dose; *I*: body shape index; *W*: body weight. Rats in each group were sacrificed at week 20.

**Figure 1:**
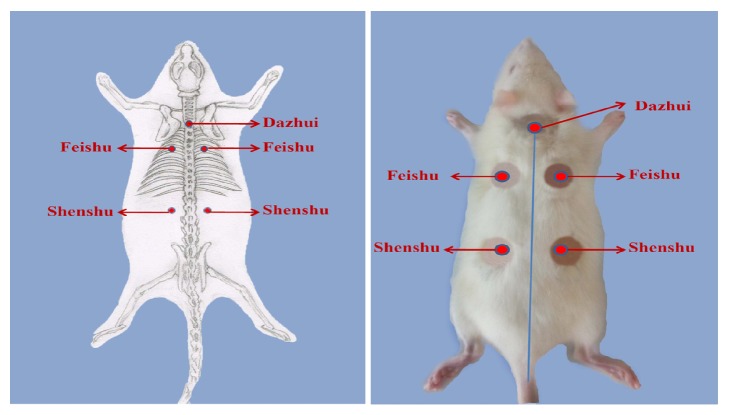
The acupoint sticking position of Dazhui, Feishu (both sides), and Shenshu (both sides).

Methods of acupoint sticking: as shown in Figure 1, the acupoint sticking was applied at Dazhui (GV14), Feishu (BL13, both sides), and Shenshu (BL23, both sides). A combination of these five acupoints can improve the lung qi and kidney qi, as well as preventing cough and asthma. The method of acupoint sticking and skin injury treatment was according to [19].

All rats were sacrificed at week 20 and samples were harvested.

### 2.4. Bronchoalveolar Lavage and Total and Differential Cell Counts

Experimental rats were sacrificed, and the left lungs were lavaged twice with 3 ml of PBS via tracheal cannulation after the right main bronchus was ligated. An equal volume of BALF was collected, and 10 *μ*l was used for total cell counts by using the “cell-count boards” method. The BALF supernatants were obtained by centrifugation (1,500 rpm  ×  10 min) at 4°C, and the samples were stored at −70°C for subsequent enzyme-linked immunosorbent assays (ELISA). The cell sediment was smeared evenly on glass slides and fixed and stained with hematoxylin-eosin. Cells were identified and differentiated into mononuclear cells, neutrophils, and lymphocytes according to standard morphology and staining characteristics. Two hundred cells per slice were quantified, and the absolute number of each cell type was calculated under a light microscope.

### 2.5. Enzyme-Linked Immunosorbent Assay

MCP-1, IL-2, IL-6, and IL-10 concentrations in BALF were quantified by using a commercial ELISA kit (RapidBio, USA) according to the manufacturer's protocol.

### 2.6. Lung Morphology

After lavage with 10% formaldehyde and fixation for 72 h, the lung tissues were cut into 3 mm thick sections, embedded in paraffin, sliced into 4 *μ*m slices, and stained with a standard method (hematoxylin-eosin) for light microscopy.

### 2.7. Immunohistochemical

For additional immunohistochemical staining, 4 *μ*m cuts were obtained. Primary antibodies against MCP-1, IL-2, IL-6, and IL-10 hydroxyguanosine (BOSTER, Wuhan, China) were used for specimen staining with the immunoperoxidase avidin-biotin method in an automatic stainer (Autostainer, Dako, Denmark). The antigen-antibody reaction was visualized with 3,3-diaminobenzidine tetrahydrochloride (DAB). Image-Pro Plus 6.0 was used for image capture and analysis. The integral optical density (IOD) represented the cytokine expression level.

### 2.8. Quantitative Real-Time PCR and Western Blotting Analysis

The expression of JNK and p38 mRNA in lung tissues was analyzed using quantitative real-time PCR (qRT-PCR). The protein expressions of JNK, p-JNK, p38, and p-p38 in lung tissue were measured by Western blotting. The methods have been described in our previous study [20]. Primers for JNK and p38 MAPK were designed and synthesized by Generay Biotech Co. Ltd. (Shanghai, China), and the sequences used in this study are shown in Table 1. 2^−ΔΔCT^ was used to calculate the changes in the relative expression of the genes in each sample.

### 2.9. Statistical Analysis

SPSS 19.0 software (IBM; Armonk, NY, USA) was used for data analysis. Data are expressed as the mean ± standard error (SE). One-way analysis of variance (ANOVA) was employed for multiple comparisons. *P* < 0.05 was considered a significant difference.

## 3. Results

### 3.1. Pulmonary Histopathological Changes

**Figure 2:**
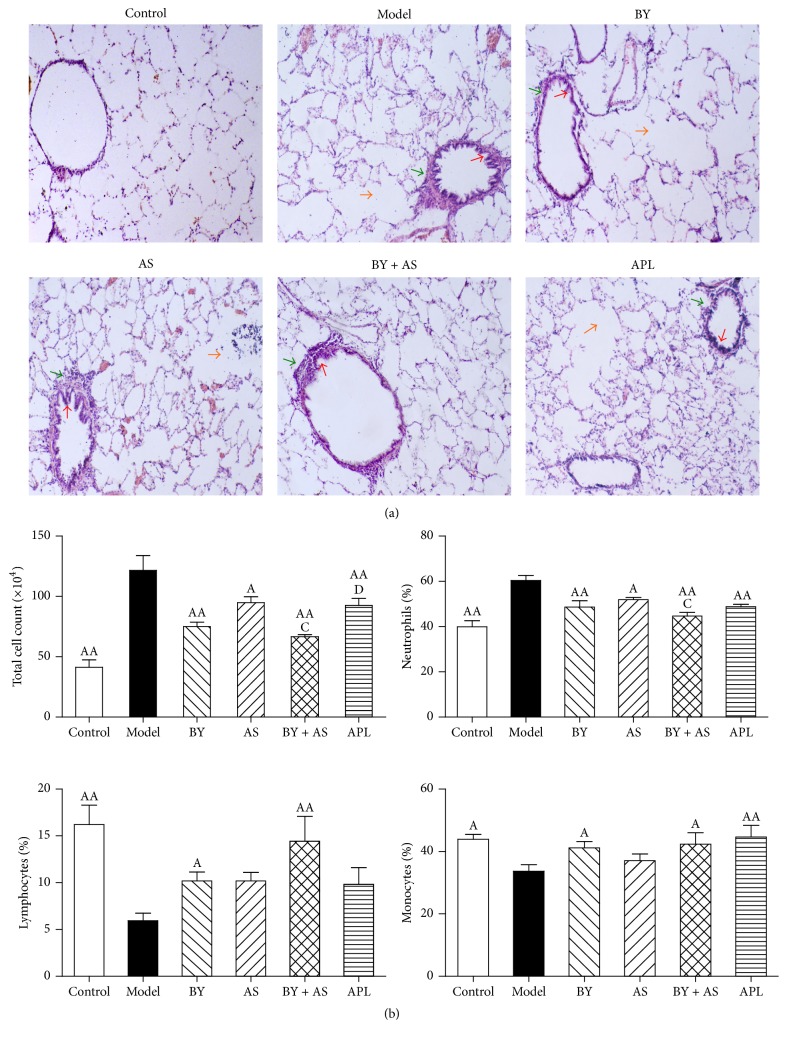
Lung morphology and total and differential cell counts in BALF of each group. Control: control group; Model: model group; BY: Bufei Yishen group; AS: acupoint sticking group; BY + AS: Bufei Yishen + acupoint sticking group; APL: aminophylline group (the same as below). Pathological changes in the lungs of each group (H&E stained ×100) (a). The orange arrows: alveolar cavity expansion; the red arrow: airway epithelial-cell hyperplasia; the green arrow: thickened small conducting airways. The total and differential cell counts in BALF (b): values are expressed as the mean ± SEM. ^AA^*P* < 0.01, ^A^*P* < 0.05 versus Model group; ^C^*P* < 0.05 versus AS group; ^D^*P* < 0.05 versus BY + AS group.

As shown in Figure 2(a), the structure of the pulmonary alveoli and airway was fully intact in Control rats. In contrast, rats in the Model group showed alterations in the submucosal and glandular tissue, including infiltration by inflammatory cells and other severe pathological changes such as epithelial-cell hyperplasia, alveolar cavity expansion, thickened small conducting airways, and connective tissue in the peribronchiolar space. Rats in the BY, AS, BY + AS, and APL groups exhibited small airway wall thickening and connective tissue hyperplasia in the peribronchiolar space, although the pathological changes were alleviated in the treatment groups to different degrees compared with the Model group, particularly in the BY and BY + AS groups. As shown in Figure 2(b), the levels of total white blood count (WBC) and the percent of neutrophils were higher compared to those in the Control group (*P* < 0.01), whereas the level of the percent of lymphocytes and monocytes was lower (*P* < 0.01 or *P* < 0.05). Compared with those in the Model group, the levels of total WBC and the percent of neutrophils in the BY, AS, BY + AS, and APL groups were significantly decreased (*P* < 0.05 or *P* < 0.01), whereas the percent of lymphocytes in BY and BY + AS groups and the percent of monocytes in BY, BY + AS, and APL groups were increased (*P* < 0.05 or *P* < 0.01). Compared with that in the APL group, the level of total WBC in the BY + AS group was decreased (*P* < 0.05). The levels of total WBC and the percent of neutrophils in the BY + AS group were decreased in comparison to the AS group (*P* < 0.05).

### 3.2. IL-2, IL-6, IL-10, and MCP-1 Levels in Bronchoalveolar Lavage Fluid (BALF)

**Figure 3:**
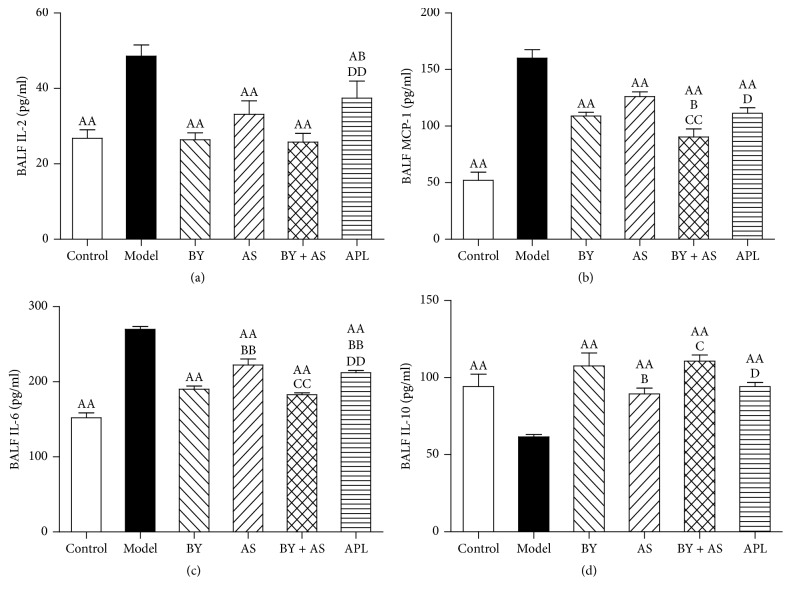
Changes in inflammatory cytokines in BALF in all treatment groups. Values are expressed as the mean ± SEM. ^AA^*P* < 0.01, ^A^*P* < 0.05 versus Model group; ^BB^*P* < 0.01, ^B^*P* < 0.05 versus BY group; ^CC^*P* < 0.01, ^C^*P* < 0.05 versus AS group; ^DD^*P* < 0.01, ^D^*P* < 0.05 versus BY + AS group.

As shown in Figure 3, the levels of IL-2, IL-6, and MCP-1 in the Model group were higher compared to those in the Control group, whereas the level of IL-10 was lower (*P* < 0.01). Compared with those in the Model group, the levels of IL-2, IL-6, and MCP-1 in the BY, AS, BY + AS, and APL groups were significantly decreased, whereas the level of IL-10 was increased (*P* < 0.05 or *P* < 0.01). Compared with those in the APL group, the levels of IL-2 and IL-6 in the BY and BY + AS groups were decreased (*P* < 0.05 or *P* < 0.01), and the level of MCP-1 in the BY + AS group was also decreased (*P* < 0.05). The levels of IL-6 in the BY and BY + AS groups were decreased in comparison to the AS group (*P* < 0.01), whereas the level of IL-10 was increased (*P* < 0.05). The level of MCP-1 in the BY + AS group was decreased compared with that in the BY group (*P* < 0.05).

### 3.3. IL-2, IL-6, IL-10, and MCP-1 in Lung Tissue

**Figure 4:**
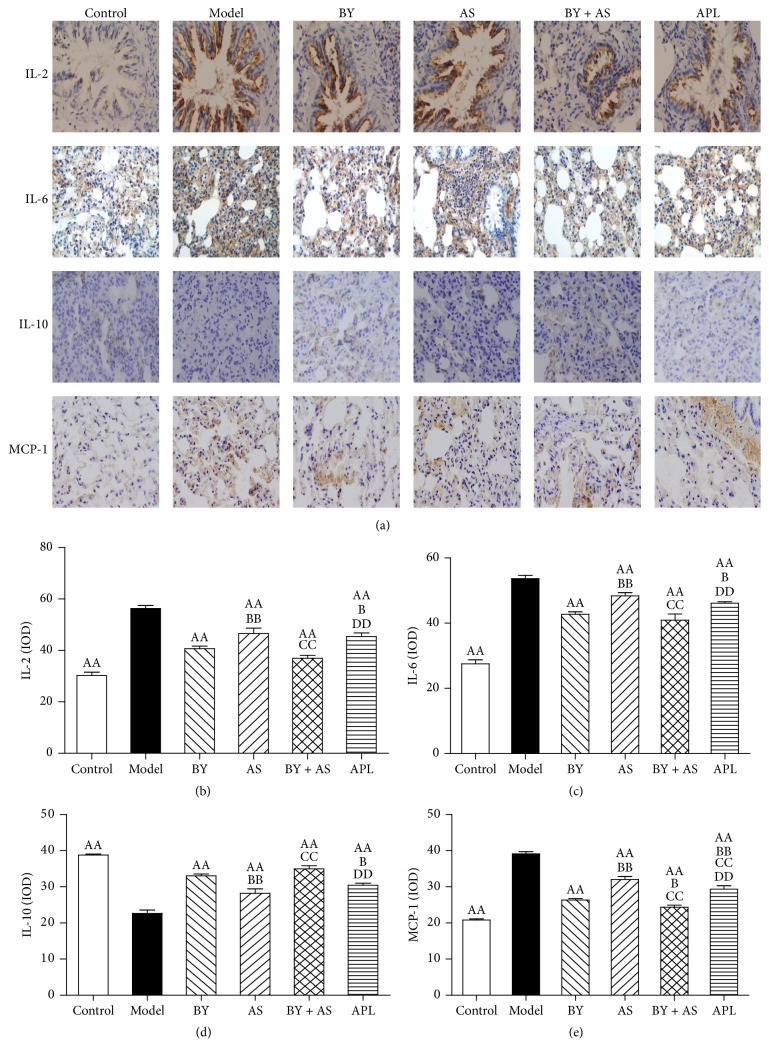
Changes of inflammatory cytokines in the lung in all treatment groups. Immunohistochemical staining of lung sections (magnification, ×400) (a); IL-2, IL-6, IL-10, and MCP-1 were quantitatively analyzed (b, c, d, and e). Values are expressed as the mean ± SEM. ^AA^*P* < 0.01 versus Model group; ^BB^*P* < 0.01, ^B^*P* < 0.05 versus BY group; ^CC^*P* < 0.01 versus AS group; ^DD^*P* < 0.01 versus BY + AS group.

As shown in Figure 4(a), IL-2 was mainly detected in the tracheal mucosal epithelium. IL-6 was distributed in the alveolar walls and alveolar interstitium, and IL-10 and MCP-1 were detected in the alveolar interstitium. As shown in Figures 4(b), 4(c), 4(d), and 4(e), the levels of IL-2, IL-6, and MCP-1 in the Model group were higher than that in the Control group, whereas the level of IL-10 was lower (*P* < 0.01). Compared with those in the Model group, the levels of IL-2, IL-6, and MCP-1 in the BY, AS, BY + AS, and APL groups were significantly decreased, whereas the level of IL-10 was increased (*P* < 0.01). Compared with those in the APL group, the levels of IL-2, IL-6, and MCP-1 in the BY and BY + AS groups were decreased, whereas the level of IL-10 was increased (*P* < 0.05 or *P* < 0.01). The level of MCP-1 in the AS group was higher than that in the APL group (*P* < 0.01). In addition, the levels of IL-2, IL-6, and MCP-1 in the BY and BY + AS groups were lower than that in the AS group (*P* < 0.01), whereas the level of IL-10 was higher (*P* < 0.01). The level of MCP-1 in the BY + AS group was lower than that in the BY group (*P* < 0.05).

### 3.4. The mRNA and Protein Expression of JNK and p38 MAPK in the Lung

**Figure 5:**
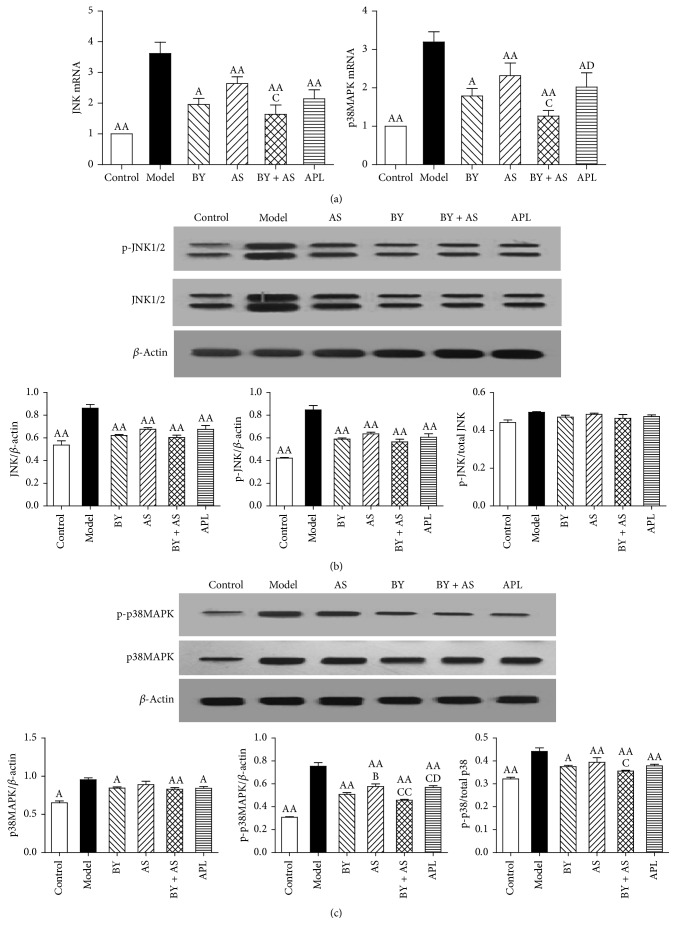
The mRNA and protein expression of JNK and p38 MAPK in the lung in all treatment groups. (a) JNK, p38 MAPK mRNA in each group; (b) the protein expression of JNK and p-JNK in each group; (c) the protein expression of p38 and p-p38 in each group. Values represent the mean ± SEM. ^AA^*P* < 0.01, ^A^*P* < 0.05 versus Model group; ^B^*P* < 0.05 versus BY group; ^CC^*P* < 0.01, ^C^*P* < 0.05 versus AS group. ^D^*P* < 0.05 versus BY + AS group.

As shown in Figure 5(a), JNK and p38 MAPK mRNA expression in the Model group was increased compared with that in the Control group (*P* < 0.01). Compared with the Model group, JNK and p38 MAPK mRNA expression in the BY, AS, BY + AS, and APL groups was decreased (*P* < 0.05 or *P* < 0.01). JNK and p38 MAPK mRNA expression in the BY + AS group was decreased compared with the AS group (*P* < 0.05), while p38 MAPK mRNA expression was decreased compared with the APL group (*P* < 0.05).

As shown in Figure 5(b), the protein expression of JNK and p-JNK in the Model group was higher than that in the Control group (*P* < 0.01). Compared with those in the Model group, the protein expression levels of JNK and p-JNK in the BY, AS, BY + AS, and APL groups were significantly decreased (*P* < 0.01). From the highest to the lowest expression, the protein level of p-JNK in each group was as follows: AS group, APL group, BY group, and BY + AS group, with no significant differences among these groups (*P* > 0.05).

As shown in Figure 5(c), the protein expression of p38 and p-p38 MAPK in the Model group was increased compared with that in the Control group (*P* < 0.05, *P* < 0.01). Compared with those in the Model group, the expression levels of p38 in the BY, BY + AS, and APL groups were decreased (*P* < 0.05 or *P* < 0.01), whereas the level of p-p38 was decreased in each group (*P* < 0.01). The protein expression of p-p38 in the BY, BY + AS, and APL groups was lower than that in the AS group (*P* < 0.05 or *P* < 0.01), although p-p38 expression was lower in the BY + AS group compared with the APL group (*P* < 0.05).

## 4. Discussion

This study was to evaluate the anti-inflammatory efficiency of Bufei Yishen granules, Shu-Fei Tie, and their combination in COPD rat model, and the results suggested that Bufei Yishen granules combined with Shu-Fei Tie therapy were beneficial for relieving lung and airway inflammation in COPD rats and that this effect was mediated via the downregulation of JNK and p38 MAPK signaling pathway.

In recent years, increasing attention has focused on the beneficial effects of TCM therapies in patients with stable COPD. The syndrome of lung-kidney qi deficiency is one of the most common syndromes in stable COPD. Our previous clinical study has confirmed the beneficial effect of Bufei Yishen granules in stable COPD [12]. Recently, we have preliminarily discussed the potential targets of Bufei Yishen granules by using systems pharmacology [23].

Acupoint sticking therapy, a kind of TCM external therapy by externally applying herbal paste to the acupoints, is popular being used for many chronic lung diseases in clinical practice. The prescription for herbal paste and the suitable acupoints are according to the intended purpose of treatment. Shu-Fei Tie, an ointment for acupoint sticking, can excite vital qi in the human body and has also been proven to be effective in preventing acute exacerbation of COPD and improving patients quality of life [17]. In our previous clinical and animal studies, Bufei Yishen granules combined with Shu-Fei Tie have been demonstrated to be beneficial in treating stable COPD; however, the mechanism responsible for these effects remains unclear.

Multiple initiating events are involved in the pathogenesis of COPD, including inflammation, protease-antiprotease imbalance, oxidant-antioxidant imbalance, and damage to the parenchyma and airways, leading to tissue remodeling. Chronic inflammation is known to play a major role in the pathological mechanism of COPD, and inflammatory cytokines, such as MCP-1, IL-2, IL-6, and IL-10, are known to promote inflammation. IL-6 is a key cytokine involved in the etiology of inflammation. Histological studies have revealed that IL-6 expression is increased in patients with COPD, and this cytokine is known to be associated with airway inflammation [24]. IL-2 is a Th1 cytokine, and inhalation of IL-2 induces asthma-like symptoms in humans and aggravates airway inflammation in a mouse model of asthma [25, 26]. IL-10 is synthesized by CD4+ or CD8+ T-lymphocytes, macrophages, monocytes, eosinophils, and the airway epithelium. IL-10 inhibits and terminates the inflammatory reaction by suppressing the synthesis and release of proinflammatory cytokines. Our study found that the levels of IL-2, IL-6, and MCP-1 in the lungs of COPD rats were increased significantly, whereas that of IL-10 was decreased. All four treatment protocols (Bufei Yishen granules, Shu-Fei Tie, Bufei Yishen granules combined with Shu-Fei Tie, and aminophylline) alleviated the expression of inflammatory cytokines in the lung and airway, whereas Bufei Yishen granules and the combined therapy showed enhanced effects compared to Shu-Fei Tie and aminophylline.

Inflammatory cytokines are mediated in part by MAPK signaling transduction pathways, such as JNK and p38 MAPK, which in turn are activated by bacterial products, cytokines, and chemokines [27]. Activation of MAPK pathways can initiate inflammatory cascades, leading to significantly increased production of inflammatory mediators such as cytokines and chemokines. Activation of the p38 and JNK pathways is involved in LPS-induced production of inflammatory molecules [28], and inhibition of the MAPK signaling pathway significantly reduces the secretion of IL-6 and IL-8 [29]. Our study showed that the mRNA and protein expression levels of JNK and p38 MAPK were increased in COPD rats. All four treatment protocols reduced the expression of these inflammatory mediators; however, Bufei Yishen granules combined with Shu-Fei Tie were more effective than Shu-Fei Tie in decreasing the expression of JNK and p38 MAPK mRNA and were more effective than aminophylline in reducing p38 mRNA. Moreover, Bufei Yishen granules combined with Shu-Fei Tie significantly decreased the protein expression of p-p38 and were more effective than Shu-Fei Tie and aminophylline. The anti-inflammatory effects of Bufei Yishen granules had been confirmed in our previous studies [13], but we did not find the overlapped anti-inflammatory effects combined with Shu-Fei Tie ointment in this study. We have found their exciting function on pulmonary surfactant proteins [30], but there may be other mechanisms involved in the function of Bufei Yishen granules combined with Shu-Fei Tie, which need our further study.

In summary, our study shows that the p38 MAPK and JNK signaling pathways are involved in regulating the expression of IL-2, IL-6, IL-10, and MCP-1 in the lung and airway in COPD rats. All four treatment protocols can alleviate lung and airway inflammation, and Bufei Yishen granules combined with Shu-Fei Tie are better than other protocols. Their anti-inflammatory effect may be involved in regulating the p38 MAPK and JNK signaling pathways.

## Figures and Tables

**Table 1:** Primer sequence of JNK and p38 MAPK mRNA.

Gene	Primer	Sequence (5′ → 3′)
GADPH	FW	ACAGCAACAGGGTGGTGGAC
	RV	TTTGAGGGTGCAGCGAAC TT
JNK	FW	TACAGAGCACCCGAGGTCATC
	RV	AGAGGATTTTGTGGCAAACCA
p38 MAPK	FW	GGC TCT GGC GCC TAT GG
	RV	CCA CAC GTA ACC CCG TTT TT

FW, forward; RV, reverse.
